# Reductive Genome Evolution from the Mother of *Rickettsia*

**DOI:** 10.1371/journal.pgen.0030014

**Published:** 2007-01-19

**Authors:** Guillaume Blanc, Hiroyuki Ogata, Catherine Robert, Stéphane Audic, Karsten Suhre, Guy Vestris, Jean-Michel Claverie, Didier Raoult

**Affiliations:** 1 Structural and Genomic Information Laboratory, Institut de Biologie Structurale et Microbiologie, Parc Scientifique de Luminy, Marseille, France; 2 Unité des Rickettsies, Faculté de Médecine, Marseille, France; Institute for Genomic Research, United States of America

## Abstract

The *Rickettsia* genus is a group of obligate intracellular α-proteobacteria representing a paradigm of reductive evolution. Here, we investigate the evolutionary processes that shaped the genomes of the genus. The reconstruction of ancestral genomes indicates that their last common ancestor contained more genes, but already possessed most traits associated with cellular parasitism. The differences in gene repertoires across modern *Rickettsia* are mainly the result of differential gene losses from the ancestor. We demonstrate using computer simulation that the propensity of loss was variable across genes during this process. We also analyzed the ratio of nonsynonymous to synonymous changes (Ka/Ks) calculated as an average over large sets of genes to assay the strength of selection acting on the genomes of *Rickettsia,* Anaplasmataceae, and free-living γ-proteobacteria. As a general trend, Ka/Ks were found to decrease with increasing divergence between genomes. The high Ka/Ks for closely related genomes are probably due to a lag in the removal of slightly deleterious nonsynonymous mutations by natural selection. Interestingly, we also observed a decrease of the rate of gene loss with increasing divergence, suggesting a similar lag in the removal of slightly deleterious pseudogene alleles. For larger divergence (Ks > 0.2), Ka/Ks converge toward similar values indicating that the levels of selection are roughly equivalent between intracellular α-proteobacteria and their free-living relatives. This contrasts with the view that obligate endocellular microorganisms tend to evolve faster as a consequence of reduced effectiveness of selection, and suggests a major role of enhanced background mutation rates on the fast protein divergence in the obligate intracellular α-proteobacteria.

## Author Summary

Genome downsizing and fast sequence divergence are frequently observed in bacteria living exclusively within the cells of higher eukaryotes. However, the driving forces and contributions of these processes to the genome diversity of the microorganisms remain poorly understood. The genus *Rickettsia,* a group of small obligate intracellular pathogens of humans, provides a fascinating model to study the genome downsizing process. In this article, we used seven *Rickettsia* genomes to reconstruct the genome of their ancestor and inferred the origin and fate of the genes found in today's species. We identify the process of gene loss as the main cause of genome diversification within the genus and show that the rate of gene loss, sequence divergence, and genome rearrangements are highly variable across the various *Rickettsia* lineages. This heterogeneity likely reflects the intricate effects of specialization to distinct arthropod hosts and critical alterations of the gene repertoire, such as the losses of DNA repair genes and the amplification of mobile genes. In contrast, we did not find evidence for the role of reduced population sizes on the long-term acceleration of sequence evolution. Overall, the data presented in this article shed new light on the fundamental evolutionary processes that drive the evolution of obligate intracellular bacteria.

## Introduction

Intracellular bacteria that are strictly associated with multicellular eukaryotes possess small genomes, typically in the range of 1 Mb or less. This feature is a consequence of the reduction of originally larger genomes invariably accompanying the adaptation to parasitic/symbiotic lifestyles. The transition from a free-living existence to a close relationship with eukaryotic cells is a frequent theme in bacterial evolution and has been documented in mycoplasmas, phytoplasmas, chlamydias, and the α- and γ-proteobacteria [1]. The *Rickettsia* genus is a group of obligate intracellular, small, rod-shaped, α-proteobacteria that possess highly reduced genomes compared to those of their free-living relatives. Known *Rickettsia* are parasites of arthropods such as ticks and insects (lice and fleas) [2,3], in which they are presumably stably maintained in the population and can be vertically transmitted. Through bites or feces of the vectors, they can infect mammals that can become sources for the next lines of infected vectors. Many members of this genus cause mild to fatal diseases in humans.

The *Rickettsia* genus provides an excellent model to investigate the process of reductive evolution. Their genomes present substantial inter-species variations in size (1.1–1.5 Mb) and gene content (about 900–1,500 genes) as a consequence of the recent and ongoing genome degradation process [4]. However, they exhibit few recent gene transfers [5] and genome rearrangements [6], which help a fine reconstruction of genome evolution history. Complete genome sequences are publicly available for five *Rickettsia* species covering the three major genus sub-groups: the typhus group (TG), including Rickettsia prowazekii (the agent of epidemic typhus transmitted by louse [4]) and Rickettsia typhi (the agent endemic typhus transmitted by flea [7]); the spotted fever group (SFG), including Rickettsia conorii (the agent of Mediterranean spotted fever transmitted by tick [5]) and Rickettsia felis (the agent of flea-borne spotted fever [6]); and the last group currently represented by a sole species, *Rickettsia bellii,* associated with ticks [8]. The availability of these five *Rickettsia* genome sequences, as well as two new SFG *Rickettsia* genome sequences determined in our laboratory (i.e., *Rickettsia africae,* the agent of African tick bite fever and *Rickettsia massiliae,* the agent of a tick-borne spotted fever in Europe [9]) prompted us to carry out a comparative genomics analysis to investigate how genome reduction and other evolutionary processes have contributed to the diversity of the genus. In this study, we identified the genes conserved in seven *Rickettsia,* inferred the gene content of their ancestors, and investigated the evolutionary dynamics of genome changes that occurred during the evolution of the genus.

## Results/Discussion

### *Rickettsia* Genes

We first built initial sets of putative genes for the seven *Rickettsia* genomes using a common protocol to avoid potential biases across the originally published data that were generated by different gene identification methods. This study aims to investigate the evolution of *Rickettsia* genomes through the reconstruction of the genome of their last common ancestor. Thus, rather than generating maximal sets of potential genes, we designed a stringent gene identification method that maximally recognizes orthologous genes that can be used to reconstruct the ancestral genome (see Methods). This method has the advantage of minimizing the inclusion of erroneously predicted genes at the cost of potentially omitting small genes that are not well supported by comparative genomics. As they probably represent a very small fraction of the genomes, we believe this potential bias does not interfere with the conclusions drawn from this study.

The predicted genes were organized into orthologous gene groups (hereafter, referred to as *Rickettsia* genes [RIGs]) based on the reciprocal best BLAST hit criterion and the extensive colinearity between the genomes. We obtained 1,867 RIGs: 1,827 for proteins and 40 for RNAs (34 tRNAs, three rRNAs, one M1 RNA, one tmRNA, and one 4.5S RNA). Genes interrupted by frameshifts or internal stop codons were flagged as pseudogenes and considered as nonfunctional. Additional pseudogenes (i.e., highly degraded gene remnants) were identified within intergenic regions using TBLASTN (see Methods). In fine, each member of the RIGs was flagged as (1) full-length gene, (2) pseudogene, or (3) absent.

The number of predicted genes in the *Rickettsia* genomes varies from 846 *(R. typhi)* to 1,324 *(R. felis)* (Table 1). Coding fraction of genome varies from 69% *(R. massiliae)* to 79% *(R. bellii).* Numbers of pseudogenes are from 100 *(R. bellii)* to 274 *(R. massiliae).* The genomes of the TG *Rickettsia* are smaller and poorer in G + C than the genomes of R. bellii and the SFG *Rickettsia* (Table 1). *Rickettsia* palindromic elements (RPEs) are interspersed repetitive sequences of 100–150 bp, abundant in both the intergenic and protein-coding regions of *Rickettsia* genomes [10] (Table S1). We found that several RPE families exhibit lineage-specific proliferation (e.g., RPE-4 in R. bellii and RPE-7 in R. felis). In all investigated *Rickettsia,* the gene encoding the phenylalanyl-tRNA synthetase β chain (PheT) has a 63- to 84-bp insertion missing in other α-proteobacteria's orthologs (Figure S1). This *Rickettsia*-specific insert exhibits a significant sequence similarity with the RPE-7 repeats. This suggests that the proliferation of this RPE family started before the divergence of different *Rickettsia* lineages.

**Table 1:**
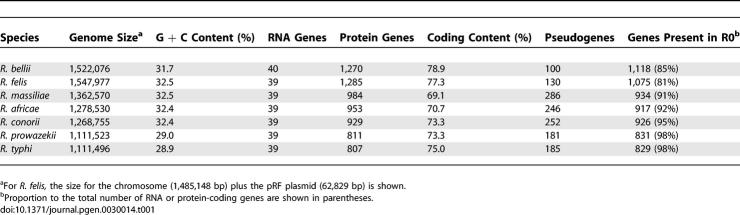
Predicted Genes in *Rickettsia* Genomes

### Core Genome Encodes Many Uncharacterized Genes

**Figure 1:**
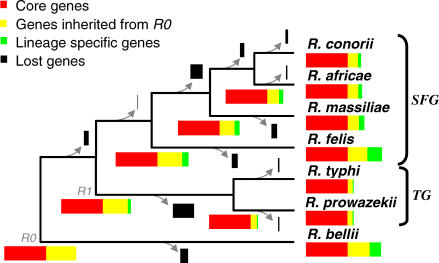
*Rickettsia* Phylogeny and Gene Contents The concatenated multiple alignment of the 704 core proteins was analyzed using neighbor joining, maximum parsimony, and maximum likelihood phylogenetic tree reconstruction methods. All methods produced the same tree topology with maximal bootstrap scores. The earliest diverging species, *R. bellii,* was chosen to root the tree [65,66]. The R0 and R1 ancestor are indicated as well as SFG and TG. The branch lengths are not to scale. Colored rectangles represent the gene repertoires of ancestral and modern *Rickettsia.* Rectangle widths are proportional to the number of genes.

The seven *Rickettsia* genomes share 704 full-length orthologous protein-coding genes and 39 RNA-coding genes. These core genes correspond to 52%–86% of the predicted gene content of each genome and reflect the functions that have been maintained in all the *Rickettsia* lineages analyzed here. Of the 704 core protein genes classified into COG (cluster of orthologous group) categories, 546 are associated with a known biochemical function. The functions of the remaining 158 genes, including 40 uniquely found in *Rickettsia,* are poorly characterized or unknown, suggesting that basic biological features shared by *Rickettsia* species remain to be elucidated. Some of the core genes are associated with the pathogenicity or parasitic lifestyle of *Rickettsia.* They include genes encoding proteins related to the virulence of *Rickettsia,* such as the hemolysins (TlyA, TlyC) [11,12], the phospholipase D (Pld) [11,13], the OmpB outer membrane protein [14], the R. conorii putative adhesin (RC1281) orthologs [15], the parvulin-like peptidyl-prolyl isomerase (SurA) [16,17], and the components of the type IV secretion system (VirB/D) [18,19]. The core gene set also includes the genes for the ATP/ADP translocases, the hallmark enzymes of intracellular parasitism in *Rickettsia* and *Chlamydia,* as well as genes related to environmental stresses such as the guanosine pentaphosphate phosphohydrolase *(gppA),* the universal stress protein *(uspA),* superoxide dismutase *(sodB),* and cold-shock protein *(cspA)* genes. The core proteins were aligned and concatenated to determine robust phylogenetic relationships between the seven species (Figure 1). This phylogenetic tree served as a reference in the following sections.

### Parasitic Ancestral Genome

**Figure 2:**
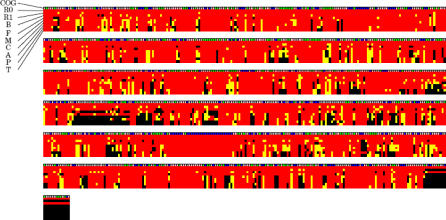
Schematic Representation of Modern and Ancestral (R0 and R1) *Rickettsia* Genomes The state of a RIG for each species or node is represented by a small box colored in red (full-length), yellow (pseudogene), or black (absent). COG classifications of the RIGs are given in the first line of the alignment: information storage and processing (blue), cellular processes (green), metabolism (pink), and poorly characterized (gray). The COG classification was assigned based on the RPSBLAST E-value of 10^−5^. The RIGs are ordered according to the inferred genome arrangement in the R1 node, except for R. bellii-specific RIGs that are displayed at the end of the alignment. Species name abbreviations are as follows: B *(R. bellii),* F *(R. felis),* M *(R. massiliae),* C *(R. conorii),* A *(R. africae),* P *(R. prowazekii),* and T *(R. typhi).* Each row of alignment corresponds to 200 RIGs.

We reconstructed gene repertoires of ancestral *Rickettsia* up to their last common ancestor, R0, using the maximum parsimony criterion (Figures 1 and 2). This reconstruction strongly supports the presence in R0 of 1,252 (1,213 protein-coding genes and 39 RNA-coding genes) out of the 1,867 RIGs. The seven *Rickettsia* genomes retained 66% *(R. typhi)* to 89% *(R. bellii)* of the 1,252 R0 RIGs (Figure 1 and Table 1), which constitute 81% *(R. felis)* to 98% *(R. prowazekii* and *R. typhi)* of the gene complement in the modern species. These figures indicate that the process of gene loss substantially contributed to the differences in gene contents between the modern *Rickettsia,* and that the TG genomes were mostly shaped by the reductive evolution from the R0 genome. The 615 remaining RIGs were specific to the R. bellii (211 cases) or the TG/SFG clades (404 cases). They may have been either (1) inherited from the R0 ancestor but lost in one clade, or (2) recently acquired by lateral gene transfer or gene duplication. Genes encoding transposases, which comprise 160 of the 615 RIGs, often occur in multiple, near-identical copies in the *Rickettsia* genomes, and, therefore, many have probably originated from recent duplications. The 65 RIGs of the R. felis plasmid have probably been acquired through a single transfer event. Among the remaining 390 chromosomal RIGs, we found only one case (*metK,* see below) of recent horizontal gene transfer that might have occurred after the divergence of R0, suggesting that many of those genes were already present in the last common *Rickettsia* ancestor. This quasi-immunity for detectable horizontal gene transfer within the *Rickettsia* genus contrasts with the many examples of lateral transfer detected prior to the R0 ancestor [8]. Our reconstruction indicates that the R0 genome contained at least 1,252, and most likely, about 1,650 genes.

Our reconstruction suggests that R0 was already parasitic, lacking most of the genes for the biosynthesis of amino acids and nucleotides and exhibiting few genes for carbohydrate degradation and transcriptional regulation. In contrast, it possessed five paralogs for ATP/ADP translocases, several genes for amino acid transporters, and the gene for RickA [20] responsible for the actin-based intracellular motility. The R0 ancestor exhibited only a few genes relevant to cofactor metabolism, among which we can cite the five full-length genes for the metabolism of biotin: a biotin synthase gene *(bioB),* a biotin (acetyl-CoA carboxylase) ligase gene *(birA),* a biotin–protein ligase gene *(bpl1),* and two genes *(bioY1* and *bioY2)* for the BioY family protein involved in the synthesis of dethiobiotin. None of the modern *Rickettsia* possesses all of the five genes in a full-length state. The R0 genome also exhibits four genes for the folate metabolism: a bifunctional folate synthesis protein gene *(folKP),* a dihydrofolate reductase gene *(folA),* a tetrahydrofolate dehydrogenase/cyclohydrolase gene *(folD),* and a gene for the 5-formyltetrahydrofolate cyclo-ligase. Of the existing *Rickettsia,* only R. bellii possesses all four genes. The R0 genome possessed a *sam* gene for uptaking S-adenosylmethionine, which is conserved in all the existing *Rickettsia* [21]. In contrast, the *metK* gene for the synthesis of S-adenosylmethionine is inferred as absent in the R0 genome, while it was inferred as full-length in R1 (the common ancestor of TG and SFG). Our phylogenetic analysis suggests that a *Rickettsia* ancestor (before R1) acquired the *metK* gene through lateral gene transfer, probably from a γ-proteobacteria (Figure S2). The *metK* gene was then degraded in different lineages of TG and SFG *Rickettsia* [22,23]. It is interesting to notice that this gene has recently been implicated in the virulence of R. prowazeki strains causing typhus [24]. Finally, the newly sequenced genome of R. massiliae exhibits a cluster of putative genes for DNA transfers, which are homologous to the *tra* gene cluster previously identified in the R. bellii genome [8]. The order of several genes flanking to the *tra* gene clusters are conserved between R. bellii and *R. massiliae.* Accordingly, our computational method predicts the presence of the 13 genes for conjugal DNA transfer in the ancestral R0 genome. Given the mobile nature of these genes, we cannot rule out that R. bellii and R. massiliae independently acquired the *tra* genes after R0.

### Dispensable Genes Vary in Their Propensity of Loss

We examined the dynamics of genome reduction process based on the computational reconstruction of the ancestral gene repertoires. Here, we considered only gene losses among the minimally predicted 1,252 R0 gene set. A gene loss event was defined as a transition from full-length to degraded (pseudogene or absent) state of a RIG in a branch of the *Rickettsia* tree. Of the 1,252 genes, 491 (39.2%) have been lost in at least one branch. Overall, we inferred 970 different gene loss events over the whole phylogeny (Figure 1 and Table S2); many genes (310, 25%) have been lost in more than one branch.

Consistent with a large variation in genome size between different *Rickettsia* species, the estimated rate of gene loss is highly variable across the branches of the tree (Figure 1 and Table S2). The R. bellii and R. felis lineages underwent fewer gene losses than the other lineages. Their genomes are the largest among the sequenced *Rickettsia.* On the other extreme, the highest number of gene losses occurred in the branch leading to the TG possessing the smallest genomes of the genus *(R. prowazekii* and *R. typhi).* The *R. massiliae, R. conorii,* and R. africae lineages went through a notable acceleration of the rate of gene loss after their separation with the R. felis lineage.

We asked if the conservation of the 743 core genes is solely due to the small number of genome samples. To test this, we used a simulation based on the model M1 (see Methods), in which 970 losses are randomly distributed among 1,252 genes, retaining the number of gene loss per branch as estimated above. The model predicts an average of 524.7 universally conserved genes (standard deviation = 8.5; 1,000 Monte Carlo samplings), which is significantly lower than the number of core genes (743) in the real data (*p* ∼0). This suggests that there has been a strong evolutionary constraint to retain at least a subset of the core genes. In other words, the gene losses were confined to a subset of the R0 gene repertoire (i.e., dispensable gene set) containing genes not essential in the context of intracellular parasitism.

**Figure 3:**
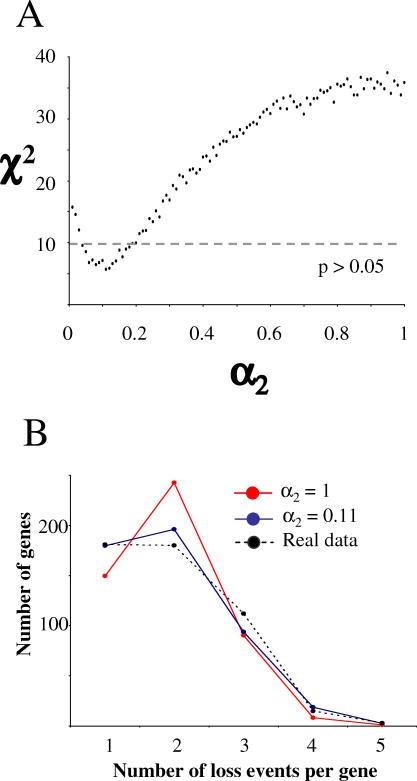
The Simulation of Gene Losses Using the Model M2 (A) Goodness of fit of the model M2 to the real data (χ2) in function of the ratio of propensity of loss α_2_. For each value of α_2_, the χ2 statistics were calculated between the distribution of the number of loss events per gene among the 491 dispensable *Rickettsia* genes and the average distribution obtained from 100 simulations with the model M2. (B) Distributions of number of loss events per gene obtained with the model M2 (averaged from 100 simulations) for selected values of α_2_.

Next, we examined the notion that dispensable genes have widely different propensities of loss [25], which may vary in function of the genome context and specialization to a new niche. We used a simulation based on the model M2 (see Methods), in which gene losses are confined to the 491 dispensable gene set. Under equal propensities of loss (i.e., M2 with *a*_2_ = 1), the simulated distribution fitted the real data poorly (*p* < 0.05; Figure 3A). If we allow two classes of genes with different propensities of loss, the distributions fitted the real data better (*a*_2_ = 0.11; *p* > 0.05; Figure 3B). This suggests that the propensity to be lost *varies across Rickettsia* dispensable genes; some genes are more prone to inactivation than others or became dispensable in the course of evolution, for instance, as a result of the adaptation to a new ecological niche.

Finally, we examined the genome reduction process in terms of lost functions and chromosomal colocalization. Genes with unknown function or general function prediction (which includes many paralogous gene families like ankyrin protein or toxin-antitoxin protein genes), as well as genes involved in defense and signal transduction, have been lost significantly more often than expected by chance (Table 2). Inversely, losses were significantly underrepresented among genes involved in protein metabolism, nucleotide and ion transport and metabolism, cellular trafficking, energy production, and cell envelope biogenesis. Colocalization of gene loss events was also obvious from Figure 2. Using the predicted initial gene order in R1 as reference (see the genome rearrangement section), we confirmed that lost genes after the R1 ancestor were more frequently clustered in the R1 genome than expected by chance (Table 3). There are two possible reasons: first, gene loss may occur through deletion of large DNA segments encompassing several genes, as for example, during the *Buchnera* genome evolution [26]. Second, genes involved in a common pathway (e.g., operon) may undergo simultaneous degradation by small-scale mutations. This can arise if a metabolic pathway becomes superfluous as a result of changes in the life cycle, relaxing the functional constraints on the underlying genes colocalized on the chromosome.

**Table 2:**
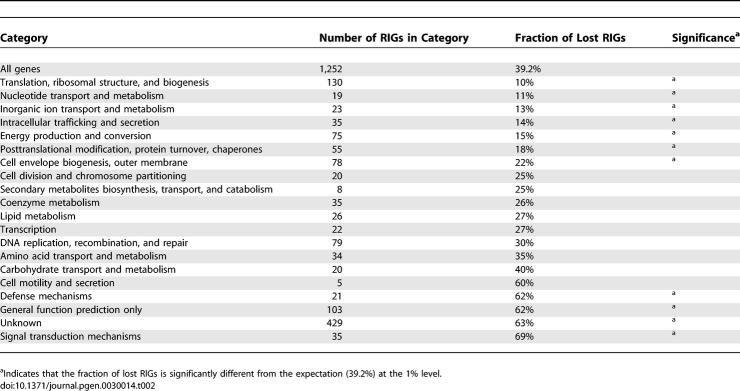
Fraction of RIGs in Different Functional Categories That Have Been Lost in at Least One *Rickettsia* Lineage

**Table 3:**
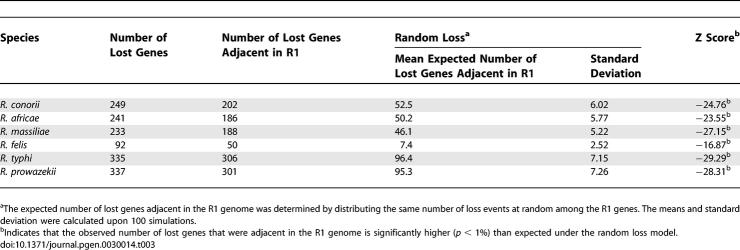
Overrepresentation of Lost Genes That Are Adjacent in the Reconstructed R1 Genome

### Faster Sequence Divergence in the Typhus Group

**Figure 4:**
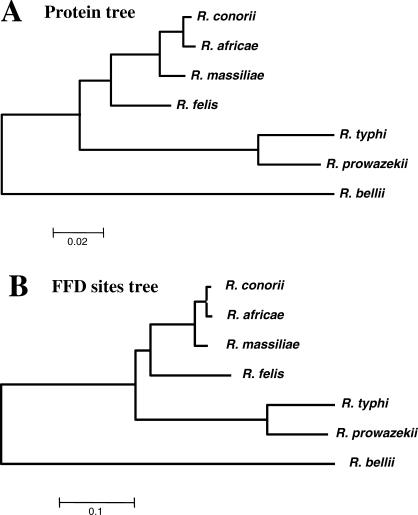
Estimation of the Branch Lengths in the *Rickettsia* Tree Using the Maximum Likelihood Method (A) Branch lengths estimated from the concatenated core protein alignment (218,887 amino acid sites) using the JTT + Γ substitution model. (B) Branch lengths were estimated from the 55,542 FFD positions of the core genes using the HKY + N2 substitution model [62].

Genome reduction correlates with an acceleration of sequence evolution [27]. We found that the TG core proteins globally diverged at a higher rate than their SFG counterparts (Figure 4A) consistent with the smaller sizes of TG *Rickettsia* genomes. The average rate for amino acid substitution was 2.43 times higher in the TG sub-tree than in the SFG sub-tree. Branch lengths estimated from the concatenated 4-fold degenerated (FFD) nucleotide positions of the core genes exhibit the same trend; i.e., the mean substitution rate at FFD sites is 2.32 times higher in the TG sub-tree than in the SFG sub-tree (Figure 4B). FFD sites are the positions in codon where any nucleotide change results in a synonymous mutation and are thus expected to be largely free from selection and to evolve at a pace similar to the mutation rate [28]. Hence, the faster protein divergence in TG is due in large part to a higher background mutation rate.

Increase of the mutation rate often correlates with the loss of genes involved in DNA repair pathways [29,30], which results in higher replication error rates [31]. Six RIGs belonging to DNA repair processes were lost in one or more *Rickettsia* (they include *phrB, radC, mutM, mutT,* and two putative alkylated DNA repair protein genes). Only *mutM, mutt,* and the alkylated DNA repair protein genes exhibit a pattern of presence/loss consistent with the higher mutation rate in TG (i.e., lost in both R. prowazekii and *R. typhi,* but retained in the other *Rickettsia*). The *Escherichia coli mutT, mutM,* and *mutY* genes protect the cell against the effects of the oxidative stress product, 8-oxoguanine, which can be incorporated during DNA synthesis and then paired with either A or C [32]. The *mutY* gene is present in all sequenced α-proteobacteria except in the order Rickettsiales (including the *Rickettsia, Wolbachia, Anaplasma,* and *Ehrlichia* genera). In *E. coli,* the *mutTmutMmutY* mutant strain produces 8.5 times more G:C to T:A transversions than the single *mutY* mutant strain [33]. These are consistent with the fast evolution and the AT enrichment for the TG genomes (*mutTmutMmutY* deficiency genotypes) relative to the SFG genomes (*mutY* deficiency genotypes) (Table 1). The variation of mutation rates among *Rickettsia* may also be due to their differences in generation times. Tick-associated SFG *Rickettsia* exhibit a longer generation time owing to their parasitic association with hard ticks that feed only two or three times during their life and have a very slow life cycle. The bacteria are in a quiescent state when ticks are not feeding and reactivated during the tick feeding [5]. In contrast, insect-associated *Rickettsia (R. prowazekii, R. typhi,* and *R. felis)* are agents of worldwide pandemics and exhibit a sporadic propagation at a very high rate by taking advantage of an arthropod-mammal cycle [3]. Thus, these bacteria may have an average generation time much shorter than tick-associated SFG *Rickettsia.*

### Similar Levels of Selective Constraints on the Proteomes of *Rickettsia* and Their Relatives

**Figure 5:**
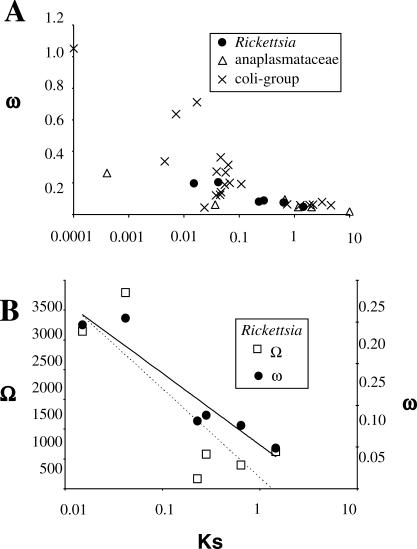
Associations between ω or Ω and Ks (A) The ratio ω = Ks/Ka and Ks were estimated between all possible pairs of genomes within a bacterial group using the concatenated alignment of 198 conserved orthologous genes. Values estimated from phylogenetically redundant pairs (e.g., R. felis and *R. conorii; R. felis* and *R. africae*) were averaged. (B) The Ω ratios (number of gene loss to Ks) were calculated between all possible pairs of *Rickettsia* genomes and close values were averaged. The number of gene loss between any two genomes was taken as the sum of the loss events inferred along the branches separating the two species (Figure 1). A logarithmic regression of Ω is represented by a dashed line. The corresponding ω ratios (same as Figure 5A) and the associated logarithmic regression (black line) are given for comparison.

In addition to a high mutation rate, the enhanced rate of protein divergence can be caused by a reduced efficiency of purifying selection [34,35]. The ratio of the level of nonsynonymous substitutions (Ka) to the level of synonymous substitutions (Ks), denoted by ω = Ka/Ks, is a classical measure of the magnitude and direction of selective constraints acting on protein sequences, with ω = 1, <1, and >1, indicating neutral evolution, purifying selection, and positive diversifying selection, respectively [36]. To compare the average selective pressures acting on the proteomes of *Rickettsia* and their relatives, we identified a set of 200 orthologous protein genes conserved in the genomes of three bacterial groups: *Rickettsia,* Anaplasmataceae, and free-living γ-proteobacteria. The Anaplasmataceae family is a group of obligate intracellular α-proteobacteria closely related to *Rickettsia* that includes the *Wolbachia, Ehrlichia,* and *Anaplasma* genera. For γ-proteobacteria, we used 13 genomes from the *Vibrio, Photorhabdus, Salmonella, Shigella,* and *Escherishia* genera (collectively referred to as the coli-group hereafter). Pair-wise ω ratios were estimated from the concatenated nucleotide alignments of the 200 conserved genes and were plotted against the level of divergence between the compared genomes measured by Ks (Figure 5A). For all of the three bacterial groups, the ω values are relatively high (ω = 0.18–0.88) for closely related genome pairs (Ks < 0.1) and decrease with increasing Ks.

It has been suggested that the obligate intracellular *Buchnera* accumulated higher fractions of nonsynonymous mutations than their free-living relatives [34,37], though this hypothesis has been recently questioned [30]. In contrast, the trajectories of ω for *Rickettsia,* Anaplasmataceae, and the coli-group converge toward comparable values (ω = 0.048–0.098 for Ks > 0.2). This suggests that, in the long term, *Rickettsia* and Anaplasmataceae retained similar proportions of nonsynonymous mutations as their free-living relatives. We cannot rule out that the convergence of ω in the three groups is due to saturation of nonsynonymous substitutions, though this is rather unlikely for comparisons with Ks < 2, as the Ka values are below 10%. Selection on synonymous codon usage in highly expressed E. coli genes can be another issue as this phenomenon tends to decrease Ks and therefore to overestimate ω. However, we could not observe any difference in ω across the three bacterial groups when strongly or weakly expressed E. coli homologs were analyzed separately (unpublished data). In summary, the elevated level of protein sequence evolution observed for *Rickettsia* and Anaplasmataceae [27,30,35] is mainly attributable to an increased background mutation rate rather than a modification of selective pressure.

### Time Dependency of the Apparent Rate of Nonsynonymous Substitution

A number of authors noted that the ω ratio is surprisingly high when very closely related bacterial sequences are compared [38–42]. Our data exhibit a similar trend (Figure 5A). The increase of ω with decreasing evolutionary distance is probably universal. As proposed by Rocha et al. [43], this time dependency of ω is probably due to a lag in the removal of nonfixed slightly deleterious nonsynonymous mutations. Sequencing errors can be another issue as their contribution to the rate estimates becomes larger when the number of true nucleotide differences is small; they bias the apparent ω toward one. However, we believe that this effect is negligible, as an independent evaluation of the sequencing error rate between two fully sequenced R. prowazekii strains indicates that less than one sequencing error occurs every 10^5^ bases on average (unpublished data). Extrapolation of our results suggests that a substantial fraction of the nonsynonymous changes observed between closely related *Rickettsia* (and other bacteria) are nonfixed mutations (i.e., polymorphisms) in their respective populations and will eventually be eliminated by natural selection.

### Time Dependency of the Apparent Rate of Gene Loss

We explored, in a similar fashion, the relationship between gene loss and level of genome divergence (Figure 5B). The number of gene losses between any two genomes was taken as the sum of the loss events inferred along the branches that separate the two species on the *Rickettsia* phylogenetic tree (Figure 1 and Table S2). We normalized the number of gene loss by Ks (hereafter referred to as the Ω ratio) in order to account for the fact that a larger number of gene losses are expected between distantly related genomes. Interestingly, the trajectory of Ω against Ks is reminiscent of that observed for ω: the number of gene loss relative to the level of synonymous changes is higher between closely related genomes and decreases with the level of divergence. A simple explanation for the dependence of Ω on the level of divergence involves the effect of polymorphism, as in the case of the elevated ω for closely related bacteria. By extrapolation, a fraction of the gene losses identified between closely related *Rickettsia* genomes may represent nonfixed slightly deleterious mutations that will eventually be eliminated from the population by natural selection (i.e., by extinction of the genotype or back mutations). This hypothesis predicts the existence of genotypes in natural populations that lack the gene loss mutations recognized in the comparisons of closely related genomes.

We investigated the presence of such polymorphisms in gene/pseudogene status using several strains and isolates of R. africae and *R. massiliae.* We selected four R. africae and five R. massiliae pseudogenes that were recently degraded in the respective lineages. By aligning the sequences of the pseudogenes with their full-length counterparts, we identified precise positions of the gene loss mutations (point mutations or insertion/deletions) causing in-frame stop codons or frameshifts in the pseudogene loci. Using PCR and sequencing, we examined the presence and absence of these mutations in different strains and isolates (five for R. africae and seven for *R. massiliae;* Table S3). We could identify one case in R. massiliae where some strains/isolates exhibited genotypes lacking the examined mutations, likely encoding a functional gene product. This suggests that genes seemingly inactivated by only a few null mutations (i.e., “split genes” [5]) may represent, in fact, polymorphic alleles and still be intact and functional in other strains of the same species.

The remaining gene loss mutations were observed in all the examined strains and isolates. It is possible that some of those mutations represent common genotypes of the species (fixed genotypes) and others are polymorphisms. In the latter case, the failure to detect genotypes lacking gene loss mutations may simply be due to our limited sample size of the strains/isolates. Further analyses of the level of polymorphism will help to quantify the time frame as well as the taxonomic scale (i.e., strain or species level) on which purifying selection acts to eliminate slightly defective genotypes from natural populations of *Rickettsia.*

### Genome Rearrangement Scenario

**Figure 6:**
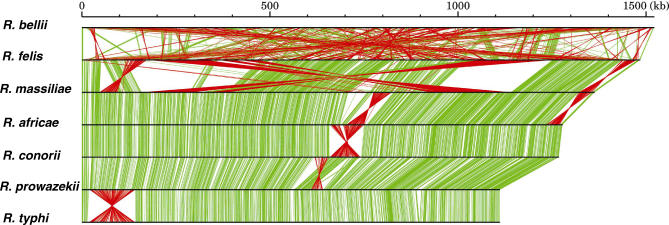
Linear Representation of *Rickettsia* Genomes The size of horizontal bars corresponds to genome size. Orthologous relationships of the genes for two adjacently aligned genomes are indicated by green (if orthologs are encoded in the same direction) or red lines (if orthologs are encoded in different directions).

Except for *R. bellii,* all other *Rickettsia* genomes exhibit long-range colinearities with solely a handful of genome rearrangements (Figure 6). We inferred a genome rearrangement (“inversion”) scenario after the divergence from the R1, using a parsimony method (Figure S3). According to the inference, zero to nine inversions (about 22–877 kb) in each branch leading to six *Rickettsia* species explain most of the genome organization differences. Half of the inversions (eight cases over 15) are symmetric to the predicted terminus of replication. Such inversions have been frequently observed [44,45]. The high genome stability of the endosymbiont *Buchnera* and the lack of horizontal gene transfer during the past 150 million y have been attributed to the loss of genes involved in DNA uptake and recombination in the initial stages of endosymbiosis [46]. In contrast, gene loss is not overrepresented among genes involved in DNA repair and recombination in *Rickettsia.* The *Rickettsia* core gene set contains orthologs for most of the genes involved in recombination in E. coli [47], including the RecF pathway, RuvABC, RecA, and part of the RecBCD system. *R. bellii, R. felis,* and R. massiliae contain a large number of nearly identical copies of transposase genes. R. bellii exhibits a highly shuffled chromosome relative to other species. The R. felis lineage also exhibits a relatively frequent chromosome inversion (nine events). Thus, the high genome stability of most *Rickettsia* is probably linked to the lack of highly similar DNA repeats rather than the loss of key genes involved in recombination. There is no correlation between the number of inversions and the number of nucleotide substitution in each branch.

### Conclusion

**Figure 7:**
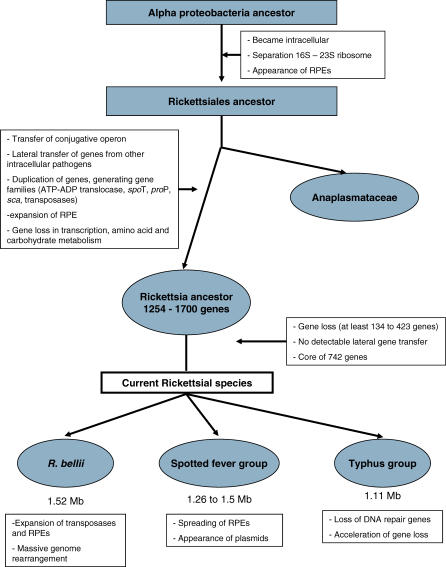
Tentative Scenario Retracing the Evolution of *Rickettsia*

With an abundance of genomic data coupled to highly conserved gene colinearity, the genus *Rickettsia* offers a convenient model for studying the phenomenon of reductive genome evolution, a common theme in obligate parasites of multicellular eukaryotes. A tentative scenario of the evolution of *Rickettsia* is given in Figure 7 and detailed below.

The ancestor of Rickettsiales, i.e., the mother of the Anaplasmataceae and Rickettsiaceae clades, was probably already a cellular parasite since all of its known descendants are obligate intracellular. It underwent a founding evolutionary event that resulted in the split of the ribosomal operon at two loci, a unique feature among these bacteria [48]. The RPE repeats may have appeared at this stage, as one of their families is present in both Rickettsiales and Anaplasmataceae [49].

After the divergence of the Anapalasmataceae, recent studies evidenced many gene transfers between the ancestors of *Rickettsiales* and intra-amoebal bacteria [8], including a conjugative operon. These bacteria probably used conjugative plasmids for gene transfer, although plasmids have been described only in a member of SFG *Rickettsia* [6]. They suggest that the rickettsial ancestor initiated intracellular parasitism in unicellular eukaryotes like amoebae, and later adapted to multicellular eukaryotes. At this stage, several gene families were expanded, such as those associated with the stringent response (*spo*T and *pro*P), membrane proteins *(sca),* and energy parasitic enzymes *(tlc)* [6]. We propose that the specialization to multicellular eukaryotes coincided with the beginning of genome reduction. The fact that the chlamydia-related obligate symbiont of amoebae has a large genome (2.4 Mb) compared to the related obligate intracellular human/animal pathogens (∼1 Mb), illustrates the consequence of this type of host transition on the genome size. In this perspective, sequencing the recently identified members of Rickettsiales living in amoeba [50] appears as a priority to better understand the early stage of genome evolution in *Rickettsia.*

The reconstructed genome of the *Rickettsia* ancestor (R0), although bigger than those of its descendants, was highly reduced (1,254–1,700 genes) lacking important biosynthesis pathways, but possessing many genes associated with parasitism. From this ancestor, we have shown that genome size differences between the modern *Rickettsia* mainly result from loss of genes presumably made dispensable in their current intracellular niche. This suggests that genome reduction is still an ongoing process in the *Rickettsia* genus [23]. As a matter of fact, we identified 100–274 pseudogenes in each genome. The massive gene loss has not been balanced by acquisition of new genes, leading to an inexorable contraction of the genome. We could not identify any recent lateral gene transfer, though paradoxically, the R0 ancestor was predicted to contain genes involved in conjugation. This could be due to the isolation of the bacteria in specific tissues of the host limiting the contact with sources of foreign genetic material. Nonetheless, the *Rickettsia* genomes underwent spreading of selfish DNAs, including lineage-specific multiplication of RPE-4 and RPE-7. Our data evidenced that RPE-7 was already present in the *Rickettsia* ancestor. Transposases were also intensely duplicated in R. felis and even more in *R. bellii.* In this study, we also demonstrated that evolutionary rates (i.e., the rates of nucleotide changes, gene losses, and chromosomal inversions) were highly variable across the different *Rickettsia* lineages. This heterogeneity likely reflects the intricate effects of specialization to distinct arthropod hosts and the critical alterations in gene repertoire, such as the losses of DNA repair genes and the amplification of mobile genes.

## Materials and Methods

### Initial gene set.

The following bioinformatic pipeline was used to build an initial gene set for every *Rickettsia:*

Step (1) For each genome, we first generated a starting dataset of open reading frames (ORFs) equal to or greater than 40 amino acids (aa) from start (ATG, GTG, or TTG) to stop codons. These ORFs were searched against the Swiss-Prot/TrEMBL sequence database (excluding *Rickettsia* sequences) [51] and the NCBI/CDD database [52] with the use of BLASTP and RPS-BLAST [53]. We also used SelfID [54] to build Markov models of genes for the *Rickettsia* genomes and generated lists of predicted protein-coding genes. ORFs shorter than 80 aa with no detectable homology (E-value < 10^−3^) in the databases were discarded. ORFs between 80 and 150 aa with no detectable homology and not identified as genes by SelfID were also discarded. Other ORFs (>150 aa) were kept.

Step (2) Pairs of ORFs overlapping by more than 30% of the size of the shorter ORF were further handled as follows: the ORF exhibiting database matches with lower E-value was kept irrespective of length; if no match was found, the longest ORF was kept.

Step (3) After identifying orthologous relationships among ORFs (see below), we further discarded the groups of orthologous ORFs shorter than 100 aa that did not exhibit detectable homology (E-value < 10^−5^) in the databases and were not present in at least two of the three major *Rickettsia* groups (i.e., SFG, TG, and R. bellii).

Step (4) Finally, we aligned orthologous ORFs to select the consensus start codons when applicable.

Step (5) tRNA genes were identified using tRNAscan-SE [55]. Other RNA genes were identified using BLASTN.

Step (6) RPEs were identified using Hidden Markov models [56] based on the previously identified RPE sequences [57].

Compared to the original annotation in GenBank, the numbers of putative protein-coding sequences are reduced in the initial gene set for four *Rickettsia* species *(R. bellii, R. felis, R. conorii,* and *R. typhi)* and increased for R. prowazekii (Table S4). The difference mostly concerns the addition or removal of small, predicted genes. Most (99%) of the predicted genes unique to the original annotations correspond to hypothetical proteins with no sequence similarity to proteins with known functions. In contrast, 64% of the predicted genes newly added in our annotation exhibit similarities to proteins with known function.

###  RIGs.

Orthologous relationships between RIGs were determined based on the reciprocal best BLASTP match criterion as well as the conservation of gene orders. Except for *R. bellii,* the *Rickettsia* genomes exhibit a nearly perfect colinearity and few genomic rearrangements. Hence, we examined and modified the orthologous relationships determined by reciprocal best matches by verifying that the order of orthologous genes were conserved in the TG and SFG *Rickettsia* genomes using the Genomeview software (S. Audic, personal communication). The orthologous gene groups were named RIGs for *Rickettsia* genes.

Next, we flagged some of the putative protein-coding sequences in the initial gene set as pseudogenes. A RIG of a species corresponding to multiple consecutive ORFs, or corresponding to a single ORF with length shorter than 50% of the size of the longest ortholog, was defined as a pseudogene. When a RIG was missing in one of the *Rickettsia* genome, we looked for gene remnants (i.e., highly degraded pseudogenes) in the corresponding orthologous genomic locus using the TBLASTN program, which performs protein against translated DNA alignments. For this search, the protein product of the longest orthologous gene was used as query. We set the E-value threshold at 0.01 and requested the size of high scoring pairs to be longer than 20 aa or 20% of the query size. This data is available in Dataset S1.

### Ancestral gene contents.

First, we inferred the gene content of the ancestral R0 genome. A RIG was considered present in the R0 genome if it was found in a full-length or pseudogene state in R. bellii and at least in one species from the SFG or TG (1,175 cases). In addition, when a RIG was present only in R. bellii or the TG/SFG clade, the protein product was searched against the GenPept database using BLASTP. The RIG was then considered to be present in the R0 genome if the score of the best match among α-proteobacteria (excluding *Rickettsia* sequences) was greater than the score of the best match against the other organisms (77 cases). Using this procedure, 1,252 of the 1,867 RIGs could be inferred as present in the R0 genome.

Of the 615 remaining RIGs, 275 RIGs exhibited a best BLAST match among organisms outside the α-proteobacteria (excluding *Rickettsia* sequences). Systematic phylogenetic analyses did not reveal any convincing case of recent horizontal gene transfer among the 275 RIGs (i.e., the *Rickettsia* proteins being anchored in a non-α-proteobacteria clade). In fact, the *Rickettsia* proteins were often the most distant sequences in the trees. The remainder (340) of the 615 RIGs did not match any sequence in database and can be therefore considered as hypothetical genes. Comparison of the GC percent at first, second, and third codon positions between the conserved 1,252 R0 genes and the 615 remaining RIGs did not reveal any significant differences.

Secondly, we determined the functional status of the 1,252 R0 RIGs in each of the ancestral nodes of the *Rickettsia* phylogeny using the maximum parsimony criterion with an irreversible transition model, which minimizes the total number of gene loss events. Concretely, a RIG was considered in a full-length state in an ancestral node if the RIG was also present in a full-length state in at least one of its descendant nodes. Otherwise, the RIG was flagged as lost (i.e., pseudogene or absent) in the ancestral node. Then, we determined the numbers of gene losses that occurred in each branch of the phylogeny by attributing a gene loss event to a branch when a RIG was inferred as full-length in the ancestral node and lost in the child node.

Finally, the ancestral states of the 615 RIGs, the presence of which could not be inferred in R0, were determined in the other ancestral nodes using the same criterion as above. A RIG was considered present in a node *i* (R1 or its descendant nodes) if it was found in a full-length or pseudogene state in at least one species for each of the two descendant clades. This data was used to generate Figure 1.

### Simulations of gene loss.

Simulations of the gene loss process were performed by distributing gene loss events among the descendants of the R0 genes along the phylogeny. For each branch, we distributed the same number of loss events as inferred from the real data. In addition, we took into account the constraints imposed by the phylogenetic context: a randomly chosen RIG could be lost along a branch if (1) it was not previously lost in the phylogenetic path up to the root (R0), and (2) it was not previously lost in any of the child phylogenetic paths. Losses of a RIG in the two branches of a bifurcation were avoided, otherwise it would be considered as a single loss in the ancestral branch according to the parsimony criterion. For a new gene loss event to be attributed to one of the available full-length genes in a branch, the probability of loss for any gene *i* was 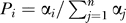 where *α_i_* is a scaling parameter that reflects the propensity of loss of gene *i* (0 ≤ *α* ≤ 1) and *n* is the number of full-length genes available for deletion. In the random model M1, we assumed equal propensities of loss among genes (α = 1 for every genes). Under model M2, the simulation of the gene loss process was carried out among the 491 genes inactivated in at least one branch of the tree. We used the same procedure as above with the additional requirement that every starting gene had to be lost at least once in the simulation. This constraint was achieved using a preprocessing step in which a first gene loss event was attributed to each RIG along a randomly chosen branch. The probability of picking a given branch was equal to the proportion of gene loss events attributed to the branch in the real data relative to the entire phylogeny. In addition, the model M2 assumed two classes of genes with different propensities of loss. The first class contained half the genes with α = *a*_1_ = 1. The second class contained the remaining genes with α = *a*_2_ ≤ 1. Note that the ratio of propensities between the two gene categories is *a*_2/_ *a*_1 =_ *a*_2._ The goodness of fit of the model M2 to the real data was measured by comparing the distribution of the average number of loss events per gene obtained from 100 simulations to the distribution observed in the real data using the χ2 statistics.

### Sequence analysis.

Protein sequences were aligned with the MUSCLE program [58]. The corresponding gene sequences were aligned using the protein alignment as a guide. We reconstructed the *Rickettsia* phylogeny using the concatenated protein alignment and three methods of phylogenetic analyses. The neighbor joining and maximum parsimony trees were constructed using the MEGA3 software [59], and the maximum likelihood tree was searched using the PHYML program [60]. All three methods recovered the same tree topology. Branch lengths were estimated using the maximum likelihood approach implemented in the PAML package [61]. For the protein datasets, we used the JTT amino acid substitution matrix and a gamma distribution to account for variable rates of substitutions among sites. Because the *Rickettsia* genomes exhibit substantial differences in GC content, we employed the nonhomogeneous HKY + N2 model of Yang and Roberts [62] to estimate the branch lengths from the FFD sites. This model accounts for unequal base frequencies. For the ω ratio analysis, reciprocal best BLASTP hits were identified between the R. conorii genome and each of the eight Anaplasmataceae, 22 coli-group, and the six other *Rickettsia* genomes. For 198 R. conorii genes, we could identify a reciprocal best BLAST hit in every target genomes. These genes were considered ortholog and aligned on a codon basis. Gapped positions in alignments were removed in order to keep only homologous codons conserved in all species. Pair-wise Ks, Ka, and ω = K_a_/K_s_ values were estimated from the concatenated alignment (55,792 codons) using the codeml program [61].

### Reconstruction of genome rearrangement scenario.

We identified colinear genome segments between the chromosomes of six species *(R. conorii, R. africae, R. massiliae, R. felis, R. prowazekii,* and *R. typhi)* based on the orthologous relationships of RIGs. The colinearity was used to represent each chromosome by a string of 27 signed characters (i.e., 1–27). The sign (plus or minus) of the characters represents the direction of the segment relative to the corresponding segment of *R. conorii.* Each character is associated with six genomic segments (from six species), of which the average size varies from 4.6 kb to 246 kb. These segments cover most (94.9% for R. massiliae to 98.8% for R. typhi) of each chromosomal sequence. We computed minimum number of inversions for each branch of the phylogenetic tree as well as the ancestral states of the strings, using these genome strings as a input for the GRAPPA release 2.0 [63] program with a fixed tree topology option. We also used GRIMM [64] for the inference of an optimal rearrangement scenario for each branch.

### PCR experiments on the gene loss mutations.

We selected four and five pseudogene loci in the genomes of R. africae and *R. massiliae,* respectively. Through sequence alignments with orthologous intact genes in closely related *Rickettsia,* we identified mutations causing pseudogenization (Table S5). We confirmed these mutations by examining the assembly of sequence reads for *R. africae* ESF5 strain and R. massiliae MTV5 strain. For each pseudogene locus, we designed a pair of primers to examine the polymorphisms in this region using five R. africae strains or seven R. massiliae strain/isolates (one reference strain, one strain growing on tissue culture, and five isolates from ticks). DNA was extracted by the QiAmp procedure (Qiagen, http://www1.qiagen.com). PCR experiments were performed using the Taq Phusion High-Fidelity DNA Polymerase (NEBiolabs, http://www.neb.com). Different elongation temperatures were tested to optimize the PCR fragments.

## Supporting Information

Dataset S1 List of *Rickettsia* Orthologous Genes (547 KB XLS)

Figure S1 Part of the Sequence Alignment of Phenylalanyl-tRNA Synthetase βchains (PheT) This alignment highlights *Rickettsia* specific inserts. Sequence parts detected as RPE-7 by a Hidden Markov model are surrounded by box. Species abbreviations are as follows: R. bellii (Rbe), R. felis (Rfe), R. massiliae (Rma), R. africae (Raf), R. conorii (Rco), R. prowazekii (Rpr), R. typhi (Rty), Agrobacterium tumefaciens (Atc), Anaplasma marginale (Ama), Ehrlichia ruminantium (Eru), *Wolbachia* wMel (Wol), and *Wolbachia* wBm (Wbm). Within the region of the *Rickettsia* specific inserts, residues that are common to at least two of the three *Rickettsia* group (R. bellii group, TG, and SFG) are highlighted by bold letters.
(33 KB PPT)

Figure S2 Phylogenetic Tree of *MetK*
The tree was constructed using the neighbor joining method implemented in MEGA with the JTT model for amino acid substitution. The sequences from the split R. prowazekii and R. africae genes were concatenated for this analysis. The tree is unrooted. Bootstrap values are indicated next to each branch.
(55 KB PPT)

Figure S3 The Most Parsimonious Scenario of Genomic Inversions in the Six *Rickettsia* Genomes (A) The numbers of inferred inversion events are indicated along the branches of the phylogenetic tree of six *Rickettsia.*
(B) A series of inferred inversions (sizes and locations) are indicated by red bars (from top to bottom according to the inferred order of their occurrence) in reference to the approximate size of the genome (green bars).
(64 KB PPT)

Table S1 Number of RPEs Found in Different *Rickettsia* Genomes (65 KB DOC)

Table S2 Number of Gene Losses and Levels of Substitutions for Branches (45 KB DOC)

Table S3 PCR Results for the Functional/Pseudogene Status of Selected Loci in R. africae and R. massiliae
(45 KB DOC)

Table S4 Comparison of the ORFs Identified in the Original Works and Those Identified in This Study (32 KB DOC)

Table S5 List of PCR Primer Sets for Testing Polymorphisms of Pseudogene Status in R. africae and R. amssiliae
(37 KB DOC)

### Accession Numbers

The GenBank (http://www.ncbi.nlm.nih.gov/Genbank) accession numbers corresponding to the *Rickettsia* genome records are as follows: R. africae (AAUY00000000), R. bellii (CP000087), R. conorii (NC_003103), R. felis (plasmid: NC_007109, chromosome: NC_007110), *R. massiliae* (AAVR00000000), R. prowazekii (NC_000963), and R. typhi (NC_006142).

The GenBank accession numbers of the Anaplasmatacea genomes are as follows: Anaplasma marginale (NC_004842), A. phagocytophilum (NC_007797), Ehrlichia canis (NC_007354), E. chaffeensis (NC_007799), E. ruminantium str. Gardel (NC_006831), str. Welgevonden (NC_005295), str. wMel (NC_002978), and Wolbachia pipientis str. wBm (NC_006833).

The GenBank accession numbers of the coli-group genomes are as follows: Escherishia coli str. CFT073 (NC_004431), str. K12 (NC_000913), str. O157:H7 VT2-Sakai (NC_002695), str. O157:H7 EDL933 (NC_002655), str. UTI89 (NC_007946), str. W3110 (NC_000091), Photorabdus luminescens (NC_005126), Salmonella enterica str. Choleraesuis (NC_006905), str. Paratyphi (NC_006511), str. typhi (NC_003198), str. Ty2 (NC_004631), S. typhimurium (NC_003197), Shigella boydii (NC_007613), S. dysenteriae (NC_007606), S. flexneri str. 2a 301 (NC_004337), str. 2a 2457T (NC_004741), S. sonnei (NC_007384), Vibrio cholerae (NC_002505), *V. fischeri* (NC_006840), str. YJ016 (NC_005139), V. parahaemolyticus (NC_004603), and V. vulnificus str. CMCP6 (NC_004459).

## Abbreviations

aa: amino acid
COG: cluster of orthologous group
FFD: 4-fold degenerated
Ka/Ks: nonsynonymous/synonymous changes
ORF: open reading frame
RIG: *Rickettsia* gene
RPE: *Rickettsia* palindromic element
SFG: spotted fever group
TG: typhus group
