# Supplementary material for: Reductive Genome Evolution from the Mother of Rickettsia
Source: PLoS Genet. 2007 Jan 19;3(1):e14. doi: 10.1371/journal.pgen.0030014 (PMC1779305; doi:10.1371/journal.pgen.0030014)
Supplement: Supplementary file 1 [file pgen.0030014.st001.doc]

**Table S1. Number of RPEs found in different *Rickettsia*** genomes.

|  | **RPE-1** | | | **RPE-2** | | | **RPE-3** | | | **RPE-4** | | | **RPE-5** | | | **RPE-6** | | | **RPE-7** | | | **RPE-8** | | |
| --- | --- | --- | --- | --- | --- | --- | --- | --- | --- | --- | --- | --- | --- | --- | --- | --- | --- | --- | --- | --- | --- | --- | --- | --- |
|  | **T** | **P** | **R** | **T** | **P** | **R** | **T** | **P** | **R** | **T** | **P** | **R** | **T** | **P** | **R** | **T** | **P** | **R** | **T** | **P** | **R** | **T** | **P** | **R** |
| *R. bellii* | 7 | 5 | - | 10 | 2 | - | 11 | 3 | - | 182 | 18 | 1 | 54 | 3 | - | 60 | 6 | - | 110 | 8 | - | 3 | - | - |
| *R. felis* | 43 | 17 | 1 | 8 | 4 | - | 17 | 6 | - | 73 | 4 | - | 63 | 4 | 2 | 105 | 8 | - | 301 | 26 | - | 33 | - | - |
| *R. massiliae* | 48 | 21 | 1 | 12 | 5 | - | 8 | 4 | - | 88 | 16 | - | 41 | - | 1 | 80 | 7 | - | 167 | 13 | - | 17 | - | - |
| *R. africae* | 47 | 20 | 1 | 11 | 5 | - | 8 | 4 | - | 83 | 12 | - | 39 | - | 1 | 80 | 8 | - | 171 | 14 | - | 21 | - | - |
| *R. conorii* | 46 | 20 | 1 | 11 | 5 | - | 8 | 4 | - | 84 | 12 | - | 40 | - | 1 | 84 | 9 | - | 167 | 14 | - | 24 | - | - |
| *R. typhi* | 12 | 7 | - | - | - | - | 5 | 4 | - | 25 | 5 | - | 26 | 2 | 1 | - | - | - | 15 | 1 | - | 8 | - | - |
| *R. prowazekii* | 14 | 9 | - | - | - | - | 5 | 4 | - | 29 | 3 | - | 25 | 2 | 1 | - | - | - | 8 | - | - | 9 | - | - |

T: total number of copies; P: number of copies within open reading frame; R: number of copies within RNA genes.

Those are the numbers of RPEs in full-length copy. Fragmented copies were not counted here.
