# Supplementary material for: Reductive Genome Evolution from the Mother of Rickettsia
Source: PLoS Genet. 2007 Jan 19;3(1):e14. doi: 10.1371/journal.pgen.0030014 (PMC1779305; doi:10.1371/journal.pgen.0030014)
Supplement: Supplementary file 2 [file pgen.0030014.st002.doc]

**Table S2. Number of gene losses and levels of substitutions for branches**

| **Branch name****‡** | **Number of Gene loss** | **Ω** | **Ka** | **Ks** | **ω** | **K_FFD_** |
| --- | --- | --- | --- | --- | --- | --- |
| RCO | 24 | 3478 | 0.0014 | 0.0069 | 0.20 | 0.005832 |
| RAF | 16 | 1702 | 0.0021 | 0.0094 | 0.22 | 0.007899 |
| AFCO | 55 | 3198 | 0.0041 | 0.0172 | 0.24 | 0.014768 |
| RMA | 63 | 3580 | 0.0044 | 0.0176 | 0.25 | 0.014605 |
| RCG | 163 | 1880 | 0.0089 | 0.0867 | 0.10 | 0.057688 |
| RFE | 85 | 615 | 0.0114 | 0.1381 | 0.08 | 0.103732 |
| SFG | 7 | 89 | 0.0061 | 0.0789 | 0.08 | 0.023764 |
| RTY | 20 | 190 | 0.0125 | 0.1055 | 0.12 | 0.099721 |
| RPR | 18 | 181 | 0.0104 | 0.0996 | 0.10 | 0.093805 |
| TG | 317 | 1112 | 0.0303 | 0.2852 | 0.11 | 0.331686 |

‡: Branches are named according to the companion figure below.

**50 gene losses**

***R. bellii***

***R. typhi***

***R. prowazekii***

***R. felis***

***R. massiliae***

***R. conorii***

***R. africae***

**AFCO**

**TG**

**RCG**

**SFG**

**RFE**

**RMA**

**RCO**

**RAF**

**RTY**

**RPR**

**RBE**
