# Supplementary material for: Reductive Genome Evolution from the Mother of Rickettsia
Source: PLoS Genet. 2007 Jan 19;3(1):e14. doi: 10.1371/journal.pgen.0030014 (PMC1779305; doi:10.1371/journal.pgen.0030014)
Supplement: Supplementary file 3 [file pgen.0030014.st003.doc]

**Table S3. PCR results for the functional/pseudogene status of selected loci in *R. africae* and *R. massiliae***.

| R. africae | **Primer set** | | | | |
| --- | --- | --- | --- | --- | --- |
| **Strains/isolates** | RAF_DEL_01 | RAF_DEL_02 | RAF_DEL_03 | RAF_DEL_04 |  |
| 12 (strain) | P | P | P | P |  |
| 15 (strain) | P | P | P | P |  |
| 16 (strain) | P | P | P | P |  |
| 21 (strain) | NA | NA | NA | P |  |
| 24 (strain) | P | P | P | P |  |
| R. massiliae |  |  |  |  |  |
| **Strains/isolates** | RMA_DEL_01 | RMA_DEL_02 | RMA_DEL_03 | RMA_DEL_04 | RMA_DEL_05 |
| K (reference strain) | F | P | P | f (3/4 examined) | P |
| 1 (strain/tissue culture) | P | P | P | P | P |
| 2 (isolate from ticks) | F | P | P | P | P |
| 3 (isolate from ticks) | F | P | P | P | P |
| 4 (isolate from ticks) | F | P | P | NA | P |
| 5 (isolate from ticks) | F | P | P | P | P |
| 6 (isolate from ticks) | F | P | P | P | P |

F: Mutations leading to a pseudogene were not observed (likely functional genes).

P: Mutations leading to a pseudogene were identified (likely pseudogenes)

f: Three out of the four potential mutations were covered by the sequence of the PCR product. No mutations leading to a pseudogene were observed for the three positions.

NA: Failures of PCR experiments.
