# Supplementary material for: Reductive Genome Evolution from the Mother of Rickettsia
Source: PLoS Genet. 2007 Jan 19;3(1):e14. doi: 10.1371/journal.pgen.0030014 (PMC1779305; doi:10.1371/journal.pgen.0030014)
Supplement: Supplementary file 4 [file pgen.0030014.st004.doc]

**Table S4.** Comparison of the ORFs identified in the original works and those identified in this study.

|  | **GenBank** | | **This study** | | **Unique in GenBank** | | **Unique in this study** | |
| --- | --- | --- | --- | --- | --- | --- | --- | --- |
|  | **No. of ORFs** | **Av. Size (aa)** | **No. of ORFs** | **Av. Size (aa)** | **No. of ORFs** | **Av. Size (aa)** | **No. of ORFs** | **Av. Size (aa)** |
| *R. bellii* | 1429 | 302 | 1395 | 307 | 34 | 77 | 0 | 0 |
| *R. felis* | 1468 | 293 | 1441 | 297 | 27 | 74 | 0 | 0 |
| *R. conorii* | 1374 | 248 | 1229 | 268 | 182 | 70 | 37 | 66 |
| *R. prowazekii* | 834 | 335 | 867 | 327 | 14 | 83 | 47 | 118 |
| *R. typhi* | 838 | 333 | 828 | 336 | 28 | 59 | 17 | 56 |

ORFs corresponding to a split gene were counted individually.
