# Supplementary material for: Reductive Genome Evolution from the Mother of Rickettsia
Source: PLoS Genet. 2007 Jan 19;3(1):e14. doi: 10.1371/journal.pgen.0030014 (PMC1779305; doi:10.1371/journal.pgen.0030014)
Supplement: Supplementary file 5 [file pgen.0030014.st005.doc]

**Table S5. List of PCR primer sets for testing polymorphisms of pseudogene status in *R. africae* (RAF) and *R. amssiliae*** (RMA).

| Primer name | Forward/Reverse primer sequences | **Intact genes in closely related species** | **Identified mutations leading to pseudogenization^*^** |
| --- | --- | --- | --- |
| RAF_DEL_01 | F:cagggaagcgtcttgatgtt  R:cctcttgcagttttgcttca | RC0799 (RIG0587) unknown | ins1, ins1 |
| RAF_DEL_02 | F:ggtacacctttgcctttatgg  R:gatccgtgcgatccaactat | RC0281 (RIG1636) capsular polysaccharide biosynthesis protein | del1 |
| RAF_DEL_03 | F:ttttagggtttattatggtgtctagtt  R:gttaatgagagcaagcgatgc | RC0076 (RIG0330) unknown | del1, stop |
| RAF_DEL_04 | F:aagcaatggtcacattacgg  R:catgagttcctatagttatacgttgtg | RC0379 (RIG1004) vapC1 | ins1 |
| RMA_DEL_01 | F:tggcaagagatagagcatcg  R:ccattcggcactggttagat | RC0146 (RIG1093) virB6 | ins1 |
| RMA_DEL_02 | F:gggaaagtaccctaccaaatca  R:aagtagcttgccacctgaaca | raf:ORF0701 (RIG0188) putP | ins2, ins29 |
| RMA_DEL_03 | F:ttagaaaacgaatttgcaacca  R:tcatggaaggccataccttt | RC0228 (RIG1137) thermostable carboxypeptidase | del600, ins1 |
| RMA_DEL_04 | F:gtgtagttacggcgggtcat  R:gcttcaacgcatatagccatc | raf:ORF1033 (RIG0236) paaJ | ins2, ins2, ins2, ins1 |
| RMA_DEL_05 | F:ttaaagaaaatgaaaagcgctac  R:cccatgattatgagcaagca | RC1031 (RIG0788) putative AAA+ superfamily ATPase | ins2, del1, ins1 |

* del*N*, ins*N* denotes a *N*-nucleotide deletion and insertion in the pseudogene region relative to the corresponding intact locus in other species. Stop denotes the presence of an in frame stop codon at the pseudogene locus.
